# Persistent Alterations of Brain and Behavior in Children With Low Prenatal Alcohol Exposure

**DOI:** 10.1016/j.bpsgos.2025.100648

**Published:** 2025-10-31

**Authors:** Xiangyu Long, Catherine Lebel

**Affiliations:** a Department of Radiology, University of Calgary, Calgary, Alberta, Canada; b Alberta Children’s Hospital Research Institute, Calgary, Alberta, Canada; c Hotchkiss Brain Institute, University of Calgary, Calgary, Alberta, Canada

**Keywords:** Alterations, Behavior, Brain, Children, Low prenatal alcohol exposure, Persistent

## Abstract

**Background:**

Heavy prenatal alcohol exposure (PAE) is associated with alterations in behavior and cognitive and brain development. However, the effects of low levels of PAE on the brain and behavior remain unclear. In the current study, we aimed to investigate longitudinal changes in the brain and behavior in children with low levels of PAE compared with well-matched unexposed children.

**Methods:**

Children (*n* = 108, mean [SD] = 9.52 [0.50] years at baseline) with PAE (0.97 ± 0.90 drinks/wk) and control children (*n* = 108, 9.52 [0.50] years at baseline) matched on socioeconomic status were selected from the ABCD (Adolescent Brain Cognitive Development) Study and were followed over 4 years with magnetic resonance imaging and Child Behavior Checklist (CBCL) scores. No children had adverse exposures to other substances.

**Results:**

Compared with unexposed children, children with low levels of PAE had persistently higher CBCL scores (worse behavior) and higher intracranial volumes over time.

**Conclusions:**

Our results provide further evidence of alterations in the brain and behavior associated with low levels of PAE across early adolescence, highlighting the importance of prevention and early intervention even with low levels of PAE.

## Plain Language Summary

Heavy prenatal alcohol exposure (PAE) is associated with changes in behavior and in the brain. However, the effects of low levels of PAE are not well understood. We studied a group of 9- to 13-year-old children with and without low levels of PAE to examine differences in the brain and behavior. Compared with well-matched unexposed children, children with low levels of PAE exhibited worse behavior and had higher brain volumes. The brain and behavior differences remained similar over time. This highlights the importance of prevention and early intervention even with low levels of PAE.

Heavy prenatal alcohol exposure (PAE) is associated with a range of adverse physical, behavioral, and neurological effects (1, 2, 3), including alterations to brain structure and function (4, 5, 6, 7). In many cases, heavy PAE can lead to a diagnosis of fetal alcohol spectrum disorder (FASD), a neurodevelopmental disorder associated with specific cognitive and behavioral difficulties estimated to affect 1% to 5% of people in North America (8, 9, 10). However, many children exposed to alcohol do not meet diagnostic criteria for FASD given that 10% to 15% of women report consuming alcohol during pregnancy in Canada and the United States (11). Therefore, there is a strong need to understand the effects of PAE outside the context of FASD (12).

The impacts of low to moderate PAE on offspring behavior and cognitive development have been investigated previously (2,13, 14, 15). However, the specific impacts of low to moderate PAE remain unclear due to many factors such as difficulty obtaining accurate measurements of alcohol consumption during pregnancy, poor control for confounding variables, and variations in experimental designs (16). For example, Sood *et al.* (17) found that 6- to 7-year-old children exposed to <9 g/day of absolute alcohol (AA) (a standard drink contains 14 g AA) during pregnancy had more aggressive and externalizing behaviors than unexposed children. Four-year-old girls with low levels of PAE (<8 g alcohol/wk) had significant mental health problems that persisted at 6 years (18). A longitudinal study found that low to moderate PAE (<14 g AA/day) was associated with internalizing and externalizing problems in young adults (19). Two recent studies using data from the ABCD (Adolescent Brain Cognitive Development) Study reported more behavioral problems in children with low PAE (20,21). On the other hand, one study showed no behavioral or cognitive differences associated with low PAE (1–2 drinks/wk) in 3-year-old children (22), and another found no relationship between 3 and 35 g AA/wk prenatally and cognition, motor, behavioral, social communication, and executive function in 6- to 8-year-old children (23). Meta-analyses have not found conclusive evidence of impacts of low to moderate PAE on social, behavioral, and cognitive functions (14,24, 25, 26), but the authors were careful to point out weaknesses of prior studies and reiterate that there is no safe amount of alcohol during pregnancy.

Beyond cognitive and behavioral deficits, a few studies have investigated the impacts of low levels of PAE on the human brain using magnetic resonance imaging (MRI). Eckstrand *et al.* (27) found smaller gray matter volumes including the left cingulate gyrus in young adults with low levels of PAE (1–7 g AA/day). Two studies using ABCD data found higher gray matter volumes (20), as well as lower fractional anisotropy (FA) (21), in 9-year-old children with low PAE. Thompson *et al.* (28) found higher intracranial volumes in children with low (≤20 g AA/occasion and ≤70 g AA/wk) to moderate (21–49 g AA/occasion and ≤70 g AA/week) PAE compared with unexposed children. These findings provide more evidence suggesting adverse effects of low and moderate PAE on neurological structure. However, it remains unclear whether these differences persist across childhood/adolescence. Using longitudinal data to evaluate how PAE affects the brain and behavior changes over time is particularly important given that longitudinal studies have shown altered brain development trajectories in children with heavy PAE (23,29, 30, 31, 32, 33).

Previously, we showed that children with low PAE from the ABCD Study had worse externalizing behaviors, higher brain volumes, and lower white matter FA compared with very well-matched unexposed control children (21). The aim of the current study was to extend our previous findings using longitudinal data. We hypothesized that the behavioral problems and brain alterations associated with low levels of PAE would extend into adolescence.

## Methods and Materials

### Participants

All participants were selected from the ABCD Study release 5.1, which includes visits from 2016 to 2022 (34). The ABCD Study was approved by a central institutional review board (IRB) at the University of California, San Diego, with local IRB approval for other sites.

Information about PAE and prenatal exposure to other adverse substances (i.e., tobacco, cannabis, heroin, cocaine, and oxycodone) was acquired at baseline (35). Participants whose biological mother answered “Yes” to using alcohol and “No” to using all other substances after knowing of pregnancy were classified as the PAE group; substance use before knowing of pregnancy was not considered in forming this group. The quantity of PAE was assessed from the answers: “Knowing of pregnancy. Average drinks per week?” and “Knowing of pregnancy. Maximum drinks in one sitting?” (Table 1). Unexposed control children were selected as participants whose biological mothers answered “No” to using any adverse substances both before and after they knew of their pregnancy and who had no diagnosis of neurodevelopmental disorders. Unexposed control children were matched to participants with PAE on age, sex, maternal education, and family income. All caregivers in both groups were biological parents. Among all ABCD participants (*N* = 11,865), 439 had parent reports of alcohol use after knowing of pregnancy. Of these, 259 participants were excluded because their parents also reported other substance use during pregnancy. In addition, 18 participants were further excluded because their parents reported high PAE (>4 drinks per week or >4 drinks in one sitting during pregnancy), leaving 162 participants with low PAE and no other substance exposure. Participants with missing information on any variable were excluded. In total, we included 108 participants with PAE and 108 matched unexposed control children at baseline (Table 1). The groups varied slightly in ethnicity, with more individuals in the PAE group identifying as White. All follow-up data available from these participants were also included.

**Table 1:** Demographics and Exposure Information for the PAE Group and the Matched Control Group at Baseline and Follow-Up

	PAE Group	Control Group	*p* Value
Baseline			
Age, years	9.52 (0.50)	9.52 (0.50)	.97
Sex, female/male	60/48	60/48	>.99
Family income, USD/year^a^	$100,000–$199,999	$100,000–$199,999	>.99
Maternal education^a^	Bachelor’s degree	Bachelor’s degree	>.99
Caregiver status	Biological mother	Biological mother	–
Ethnicity			.01
African American	5	8	
White	99	84	
Mixed	4	16	
Knowing of pregnancy			
Maximum drinks in 1 sitting, *n* = 106	1.01 (0.37)	NA	NA
Average number of drinks per week, *n* = 104	0.97 (0.90)	NA	NA
Other exposure to adverse substances	None	None	
1-Year Follow-Up			
Age	10.50 (0.61)	10.55 (0.61)	.54
Sex, female/male	57/47	58/48	
2-Year Follow-Up			
Age	11.60 (0.74)	11.63 (0.74)	.78
Sex, female/male	59/48	56/46	
3-Year Follow-Up			
Age	12.47 (0.72)	12.47 (0.65)	>.99
Sex, female/male	56/42	55/44	
4-Year Follow-Up			
Age	13.78 (0.85)	13.80 (0.75)	.86
Sex, female/male	26/27	28/23	

Values are presented as mean (SD) or *n*.

PAE, prenatal alcohol exposure.

a Family income and maternal education are the median levels across participants for each group. Groups were well matched on all relevant demographic and socioeconomic factors.

For complementary analyses, 2 more comparison groups were selected (more details are provided in the Supplement). The first comparison group was participants (*n* = 187) who were prenatally exposed to tobacco without other adverse substances (i.e., alcohol, cannabis, other drugs); this is the tobacco-only group. The second comparison group included participants (*n* = 108) who were not prenatally exposed to any adverse substances and had age, sex, parental educational levels, family incomes, and Child Behavior Checklist (CBCL) scores similar to the PAE group.

### Behavior Measurements

The CBCL, including the DSM-5–oriented scores, was used to assess child behavior. The CBCL is a parent-report instrument (35, 36, 37). The CBCL provides internalizing, externalizing, and total problems summary scores, as well as scores of anxious/depressed, withdrawn/depressed, somatic complaints, social problems, thought problems, attention problems, rule break, sluggish cognitive tempo, obsessive compulsive, stress, and aggressive behavior. The DSM-oriented CBCL scores provide measures of depression, anxiety, somatic, attention-deficit/hyperactivity (ADHD), oppositional-defiant, and conduct disorders. Behavior assessments were done annually and were available for the PAE group and the matched control group at 5 time points (Table S1).

### Neuroimaging Data

Tabulated MRI measurements from the ABCD Study were selected (34), including regional gray matter volumes (193 items), surface area (151 items), sulcal depth (151 items), and cortical thickness (151 items); full shell diffusion tensor imaging FA (181 items) and mean diffusivity (MD) (181 items) within subadjacent white matter regions and deep brain areas; FA (42 items), MD (42 items), and volumes (42 items) of the atlas-based fiber tracts; and temporal variance of functional MRI (fMRI) time series (181 items) and functional connectivity (416 items). The Destrieux atlas was used to define brain regions (38). Preprocessing details can be found in Hagler *et al.* (39). Three time points of imaging data were analyzed. Multisite imaging data harmonization was conducted by ComBat for each visit separately (40,41).

### Statistical Analysis

A linear mixed-effects model was applied to the whole sample across all visits using the following equation:

(1) CBCLmeasures=Intercept+β1×Groups+β2×Age+β3×Sex+β4×Groups×Age+β5×(1|Subjects)

(2) MRImeasures=Intercept+β1×Groups+β2×Age+β3×Sex+β4×Intracranialvolumes+β5×Groups×Age+β6×(1|Subjects)

If the coefficient for the age-by-group interaction was not significant, the interaction term was dropped from the model, and the model was rerun with only main effects. The coefficients were tested and corrected using false discovery rate (FDR) correction at *p* < .05 across all CBCL or MRI measures separately.

A linear mixed-effects model was applied to the MRI metrics and CBCL scores at baseline and 2-year- and 4-year follow-up visits for each group based on the significant findings from the previous mixed-effects model analyses:

(3) CBCL=Intercept+β1×MRImeasures×Groups+β2×MRImeasures+β3×Groups+β4×Age+β5×Sex+β6×(1|Subjects)

The coefficients for MRI-by-group interactions were tested and corrected using an FDR-corrected *p* < .05 across all CBCL and MRI measures combinations.

## Results

### Alcohol Consumption

In the PAE group at baseline, the maximum number of drinks reported in one sitting was 1.01 (0.37), and the average number of drinks per week in pregnancy was 0.97 (0.90) (Table 1). Similar amounts of PAE were reported in the follow-up years (1.02 maximum drinks/sitting and 0.98 average drinks/wk). No alcohol consumption was reported in the unexposed control group.

### Behavioral Scores

**Figure 1:**
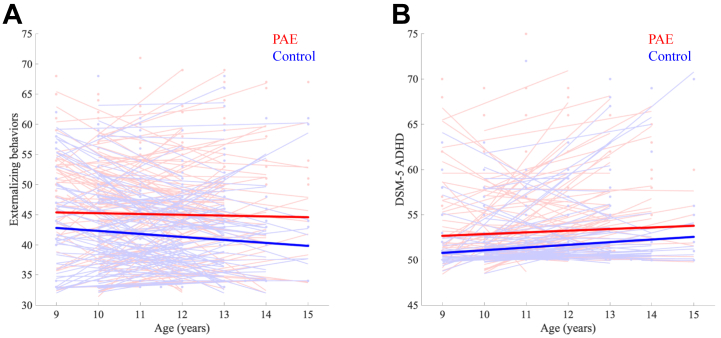
Child Behavior Checklist **(**CBCL)/DSM-5 scores over time. **(A)** Externalizing scores for the prenatal alcohol exposure (PAE) group (red), compared with the unexposed control (blue) group, across all visits show persistently higher scores, indicating worse problems. **(B)** The DSM-5 attention-deficit/hyperactivity disorder (ADHD) scores for PAE (red) and unexposed control (blue) groups across all visits similarly indicate worse problems over time in the PAE group. Each light color trend line in panels **(A)** and **(B)** represents 1 participant across visits. Bold red and blue lines are trend lines for each group.

No behavioral scores showed significant age-by-group interactions, so interaction terms were dropped from all models. For main effects, the PAE group had higher CBCL scores (i.e., more problematic behaviors) than the control group in several domains (Table 2, Figure 1), including worse attention problems, internalizing behavior, externalizing behavior, total problems, and DSM-5 ADHD scores. Despite this, most participants with PAE (∼90%) had scores in the normal range (i.e., <65).

**Table 2:** Mean ± SD Scores and Group Differences on CBCL and DSM-5 Measurements Across All Visits

CBCL^a^	PAE	Control	95% CI	*p* Value	Cohen’s *d*	PAE, % With Score ≥65	Control, % With Score ≥65
Anxious/Depressed	54 ± 6	53 ± 5	−0.30 to 2.17	.061	0.2	7.46%	5.59%
Withdrawn/Depressed^b^	54 ± 6	52 ± 4	0.33 to 2.47	.002	0.3	7.25%	3.23%
Somatic Complaints	55 ± 6	54 ± 6	−0.04 to 2.50	.089	0.2	9.17%	6.67%
Social Problems^b^	52 ± 4	51 ± 2	0.20 to 1.64	.012	0.3	2.35%	0.43%
Thought Problems^b^	54 ± 5	53 ± 4	0.12 to 2.13	.014	0.2	6.18%	1.94%
Attention Problems^b^	54 ± 5	52 ± 3	1.12 to 3.00	<.001	0.5	5.54%	1.08%
Rule Break^b^	52 ± 3	51 ± 2	0.24 to 1.40	.003	0.3	1.49%	0.22%
Aggressive^b^	52 ± 4	51 ± 3	0.14 to 1.71	.012	0.3	2.77%	1.29%
Internalizing^b^	49 ± 11	47 ± 10	0.31 to 4.85	.016	0.3	8.53%	5.59%
Externalizing^b^	45 ± 9	42 ± 8	1.31 to 5.23	<.001	0.4	2.35%	0.65%
Total Problems^b^	46 ± 11	42 ± 9	1.17 to 5.77	.002	0.4	3.20%	1.29%
DSM-5_Depression^b^	54 ± 6	53 ± 5	0.33 to 2.78	.004	0.3	8.96%	6.02%
DSM-5_Anxiety	54 ± 6	53 ± 6	−0.58 to 1.98	.162	0.1	7.46%	6.67%
DSM-5_Somatic	56 ± 7	55 ± 6	−0.02 to 2.76	.079	0.2	11.30%	7.74%
DSM-5_ADHD^b^	53 ± 5	51 ± 3	0.77 to 2.57	<.001	0.4	5.12%	1.29%
DSM-5_Oppositional-defiant^b^	53 ± 5	52 ± 3	0.60 to 2.38	.001	0.4	4.05%	1.51%
DSM-5_Conduct^b^	52 ± 4	51 ± 3	0.10 to 1.50	.013	0.3	2.35%	0.86%
Sluggish Cognitive Tempo^b^	53 ± 6	51 ± 3	0.79 to 2.92	<.001	0.4	6.40%	0.86%
Obsessive Compulsive	54 ± 5	53 ± 5	−0.10 to 2.15	.043	0.2	4.26%	4.52%
Stress^b^	53 ± 5	52 ± 4	0.24 to 2.29	.008	0.3	5.12%	2.15%

ADHD, attention-deficit/hyperactivity disorder; CBCL, Child Behavior Checklist; PAE, prenatal alcohol exposure.

a All CBCL scores are T scores.

b These rows indicate domains that survived false discovery rate correction at *p* < .05.

### Neuroimaging Metrics Associated With PAE

**Figure 2:**
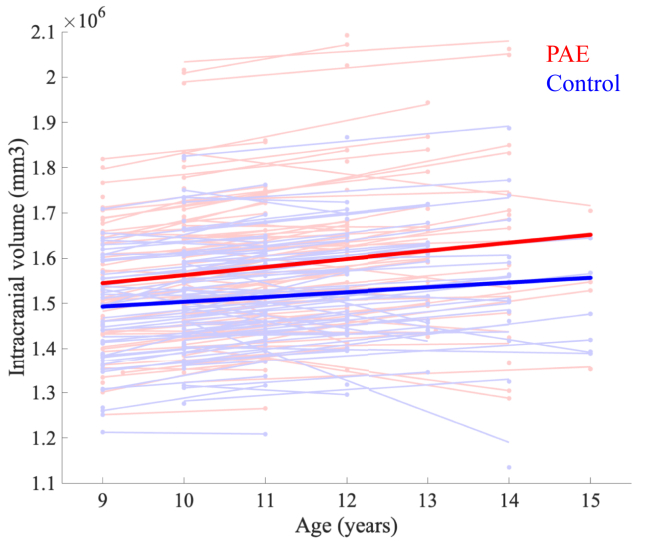
Intracranial volume. Intracranial volume for prenatal alcohol exposure (PAE) (red) and unexposed control (blue) groups across all visits shows persistently higher volumes in the PAE group. Each light color trend line represents 1 participant across visits. Bold red and blue lines are trend lines for each group.

None of the imaging metrics had significant age-by-group interactions, so this term was dropped from all models. For main effects, adolescents with low levels of PAE had higher intracranial volumes and other total brain volume measures (left, right, and total cortical gray matter volumes) compared with the unexposed control group (Table 3, Figure 2).

**Table 3:** Significant Group Differences (Prenatal Alcohol Exposure − Control) on the Magnetic Resonance Imaging Measurements Across Visits

Measures	Brain Area	95% CI	*p* Value	Cohen’s *d*
Cortical Volume	Left hemisphere Destrieux regions in total	3593.21–10,876.29	<.001	0.6
Cortical Volume	Right hemisphere Destrieux regions in total	3559.33–10,825.10	<.001	0.5
Cortical Volume	All Destrieux regions in total	7309.80–21,871.91	<.001	0.5
Volume	Intracranial	95,368.03–29,105.48	<.001	0.4
Volume	Supratentorial	15,956.09–47,287.85	<.001	0.5

Significant differences at *p* < .05 after false discovery rate correction.

### Associations Between CBCL Scores and MRI Metrics

No associations between CBCL scores and MRI metrics survived FDR correction.

## Discussion

To our knowledge, our study is the first to show persistent brain and behavior differences associated with low levels of PAE in youth. These results extend previous findings at the baseline ABCD time point of 9 years (21), suggesting that brain alterations and behavioral problems associated with low levels of PAE are stable across early adolescence and are primarily global in nature.

### Problematic Behaviors Associated With Low PAE

Heavy PAE has been consistently associated with increased problematic behavior across multiple domains (42, 43, 44, 45). Some studies have shown that lower PAE is also related to behavior problems (46,47), including a study by Lees *et al.* (20) that reported problematic behaviors at both the baseline year and 1-year follow-up in the ABCD cohort. In our previous study of ABCD baseline data, only externalizing behaviors were significantly worse in children with low levels of PAE compared with matched unexposed control children (21). Here, using more time points and better data quality control (see the ABCD release notes: doi: 10.15154/z563-zd24 for more details), we showed effects across a wider range of behaviors. Of particular importance is the fact that none of these domains showed significant moderations by age, suggesting that at a group level, low-level PAE increases behavioral problems and these problems persist across early adolescence. It is worth noting that most participants with PAE had CBCL scores within the normal range, suggesting that low PAE increases subthreshold mental health symptoms but results in clinically elevated symptoms only for a small number of individuals. This also demonstrates the heterogeneity of the impacts of PAE and highlights the need for more research to understand the specific factors driving vulnerability or resilience.

### Brain Changes Associated With Low PAE

In addition to worse behavior scores, we found higher intracranial volumes in youth with low levels of PAE compared with unexposed control youth. Increased brain volumes at the baseline year are consistent with 2 previous studies in the ABCD dataset (20,21), as well as a separate cohort of children with low to moderate PAE showing higher intracranial and bilateral caudate volume (28). However, these findings contrast with most prior studies of heavier PAE and FASD (4,6,7,48), with a few exceptions (49). These findings suggest that low levels of PAE may act on the brain in a different way than heavy PAE, manifesting seemingly opposite effects on the child and adolescent brain. Previous studies have found that brain morphometrics show a linear dose-dependent relationship with alcohol during pregnancy wherein higher alcohol exposure is associated with decreased gray matter volumes (27) or thinner cortex (50). However, prior work has generally excluded individuals with low levels of PAE. Therefore, our results suggest that there may be nonlinear effects of PAE dosage, where low PAE is associated with higher brain volumes, and after a certain threshold, higher PAE is associated with progressively lower brain volumes. This requires future studies with participants across a range of well-characterized PAE.

The mechanisms underlying increased brain volumes associated with low levels of PAE remain unclear. Brain overgrowth has been reported in other neurodevelopmental disorders such as autism, although the mechanism is also unclear (51). Typical brain development involves substantial reductions of gray matter volume across adolescence, thought to reflect synaptic and neuronal pruning (52,53); therefore, delayed (or impaired) synaptic and neuronal pruning related to PAE would result in higher volumes, although the persistent nature of differences here does not suggest delayed maturation. Increased myelination of cortical gray matter during development (54) could potentially be a compensatory response to counter the adverse effects of PAE and would also lead to larger volumes (20,49). Future longitudinal studies are needed to further understand the observed increased brain volumes in participants with low amounts of PAE, especially across a range of ages and exposures.

Prior studies have reported altered white matter microstructures in children with PAE, primarily lower FA and/or higher MD (5,55), although there are exceptions (56, 57, 58). Notably, despite data updates and a different sample size, some findings from our prior study at ABCD baseline were similar in the current study. Lower FA in the left postcentral area was found at baseline in our prior study without controlling for intracranial volume (21); current data show lower FA in the right postcentral area (95% CI, −0.01 to 0.00; *p* = .045, uncorrected) while controlling for intracranial volume. However, the FA findings in the current study did not survive multiple comparison correction and thus suggest that white matter microstructural metrics may not be as sensitive to low PAE as global brain volume differences.

Alterations in intracranial volume remained stable across the age range. The small but growing literature of longitudinal MRI studies of PAE has shown somewhat contrasting results. Some studies have reported significant group-by-age effects on cortical volume (31), cortical thickness (59), and white matter microstructure (58,60), while other longitudinal studies have shown relatively similar trajectories in children with and without PAE. For example, one longitudinal study found few group-by-age interactions, and only white matter volume of the right and left transverse temporal regions showed differing trajectories between groups (61). A longitudinal study with scans 20 years apart found no group-by-age interactions in brain volumes in adults with PAE (62). Another longitudinal study found reduced thalamus volumes in children and youth with PAE at 2 longitudinal visits that were 2 to 4 years apart, with no group-by-age interactions (29). All previous longitudinal studies have been with individuals with heavy PAE, who may have more widespread and/or more profound effects on both brain structure and its changes over time than the children with low levels studied here.

To help further understand these differences, we performed 2 complementary analyses comparing the PAE group with participants with tobacco exposure only (no alcohol or other substances) and comparing the PAE group with the unexposed control group with matched CBCL scores. In both analyses, the PAE group had significantly higher intracranial volumes than the comparison group (see details in the Supplement). This suggests that the finding of increased intracranial volumes is not a general finding common to behavior problems or any prenatal substance exposure and is rather (at least somewhat) specific to PAE.

No brain differences were detected in fMRI measures such as signal variance or functional connectivity. Previous studies have reported atypical brain functional network efficiency and altered intra-/internetwork connectivity in children with heavier PAE (63, 64, 65, 66). While this suggests that low levels of PAE may not impact brain function as much as high levels of PAE, the worse behavior scores found here suggest that brain function is not necessarily spared from the effects of low PAE. Further studies may be needed to understand the potentially nuanced effects of low PAE on brain functional metrics.

### Relationships Between Behavior and Brain Metrics

There were several interactions between CBCL scores and group, although none of them survived multiple comparison corrections due to the high number of tests (34,620). Previous studies have reported disrupted brain-behavior relationships associated with PAE (21,57,67,68). For example, one study found positive relationships between right anterior midcingulate cortex volume and internalizing scores in children with PAE but not in the control group (69). In our previous study, white matter FA had negative relationships with CBCL scores in the unexposed control group but not in the PAE group (21). These disrupted relationships are important to consider when designing and evaluating interventions and may be apparent in larger samples of participants with lower levels of PAE or with more targeted tests.

### The Importance of Selecting a Well-Matched Control Group

Numerous factors can impact behavior and brain structure/function, including age, sex, and sociodemographic variables. Lower socioeconomic status (SES) is associated with increased internalizing and externalizing psychopathology during childhood (69, 70) and differences in internalizing/externalizing problems between females and males (71). This is also true in studies of PAE, which have shown interactions between PAE, sex, behavior, SES, and brain metrics (33,72). A substantial advantage of the current study is that the unexposed control group was matched to the PAE group on age, sex, SES, caregiver status, and lack of other substance exposure. Furthermore, additional control groups showed that the increased brain volumes were not related to tobacco exposure or behavior scores. This careful matching helps mitigate the potential impacts of other factors on our findings and gets closer to establishing PAE as the cause of the differences. While the current study cannot establish causality, it can make a stronger link between PAE and behavior and brain alterations than prior work with less well-matched control groups.

### Implications for Policy Associated With Drinking During Pregnancy

The average exposure in this study was only 1 drink/wk, suggesting that even low levels of drinking during pregnancy can have lasting negative effects and should be considered in prenatal care settings. While medical groups in North America already recommend no alcohol exposure during pregnancy, many prenatal care providers believe that small amounts of alcohol are safe (73). The current findings could help support the recommendations from the Society of Obstetricians and Gynaecologists of Canada, Centers for Disease Control and Prevention, and World Health Organization, as well as other groups, to abstain from alcohol entirely during pregnancy (74,75) and help care providers understand that even low PAE is associated with negative outcomes. This work also highlights the importance of recognizing and providing support for children with any level of PAE who have behavioral and/or learning challenges.

### Limitations

Prenatal exposure information was collected using retrospective questionnaires that biological mothers completed when their children were 9 years old. This may impact the accuracy of PAE quantities, although previous studies have successfully used retrospective questionnaires (76). Furthermore, the anonymous questionnaires used in the ABCD Study could ameliorate the accuracy and honesty of alcohol reporting (35), and having biological maternal reports of PAE is a strength of this study, as many prior studies of PAE have included children without providing details of the quantity and frequency of PAE. Another limitation is that not all participants from baseline completed longitudinal data collection, resulting in a reduced sample size at later years. We overcame this using a mixed-effects model analysis; however, future studies with better retention will help understand the long-term effects of PAE in greater detail.

### Conclusions

Our results provide strong evidence of persistent alterations in brain and behavior associated with low levels of PAE. Compared with well-matched unexposed control youth, youth with low levels of PAE had worse attention, depression, and conduct scores, as well as increased intracranial volumes from 9 to 13 years. These adverse effects of low PAE are sustained rather than mitigated over later childhood and early adolescence, highlighting the importance of prevention and early intervention even for children with low levels of PAE. Given that approximately 10% of individuals report drinking during pregnancy, and many prenatal health care providers do not convey advice to abstain (73), there is a clear need for better prevention and intervention associated with low levels of exposure. These results have important implications for policy recommendations to support health care providers and public health messaging to convey clear advice and to support individuals who want to cease alcohol consumption during pregnancy.

## Supplementary Material

Supplemental Methods, Results, Tables S1–S2

Key Resources Table
