# Supplementary material for: Persistent Alterations of Brain and Behavior in Children With Low Prenatal Alcohol Exposure
Source: Biol Psychiatry Glob Open Sci. 2025 Oct 31;6(2):100648. doi: 10.1016/j.bpsgos.2025.100648 (PMC12804615; doi:10.1016/j.bpsgos.2025.100648)
Supplement: Supplemental Methods, Results, Tables S1–S2 [file mmc1.pdf]

SUPPLEMENTARY INFORMATION

Persistent Alterations of Brain and Behaviour in Children With Low Prenatal Alcohol Exposure

Long and Lebel

Supplementary materials

Results of including interaction term groups × sex

Additional analyses were performed with an additional term on the interaction between groups and biological sex:

CBCL measures = Intercept + β1 × Groups + β2 × Groups × Sex + β3 × Age + β4 × Sex + β5 × (1 | Subjects)

Intracranial volumes = Intercept + β1 × Groups + β2 × Groups × Sex + β3 × Age + β4 × Sex + β5 × (1 | Subjects)

No significant effect was detected in the groups × sex term on both CBCL scores and intracranial volumes ($t = -1.51$, $p = 0.132$) after FDR correction. Only withdrawn/depressed scores ($t = 2.24$, $p = 0.025$) and DSM5 depression scores ($t = 2.08$, $p = 0.038$) showed sex effects before FDR correction.

Complementary analyses

Two more comparison groups were selected to detect the effects associated with PAE (Table S1). The first group was the participants (n = 187) who prenatally exposed only to tobacco without other adverse substances (i.e., alcohol, cannabis). The second group was a control group (n = 108) who was not prenatally exposed to any adverse substances, and had similar age, sex, parental educational levels, family incomes and CBCL scores to the PAE group.

Table S1. Demographics for the adolescents with prenatal tobacco exposure and CBCL-matched controls (with the PAE group) at baseline and follow-up.

		Tobacco exposure	CBCL matched controls
Baseline	Age (mean ± std year)	9.46±0.5	9.5±0.5
	Sex (female/male)	105/82	47/61
	Family income (median, USD/year)	$35,000 through $49,999	$100,000 through $199,999
	Maternal education (median)	Some college	Bachelor's degree
	Caregiver status	Biological mother	Biological mother
1-year follow-up	Age (mean ± std)	10.41±1.04	10.5±0.62
	Sex (female/male)	98/76	44/59
2-year follow-up	Age (mean ± std)	11.54±0.69	11.93±3.14
	Sex (female/male)	94/71	45/60
3-year follow-up	Age (mean ± std)	12.36±1.24	12.49±0.69
	Sex (female/male)	83/61	38/58
4-year follow-up	Age (mean ± std)	13.47±1.75	13.72±0.63
	Sex (female/male)	44/31	18/25

Results of the complementary analyses

The tobacco-only group showed lower parental educational levels ($t = -12.98$, $p < 0.001$) and family incomes ($t = -13.31$, $p < 0.001$) than the PAE only group, so we controlled for these in analyses. The tobacco-only group showed significant smaller intracranial volumes ($t = -3.55$, $p < 0.001$) than the PAE-only group when controlling for age, sex, parental educational levels and family incomes. There was no difference on CBCL scores between the tobacco-only group and the PAE-only group after controlling for age, sex, parental educational levels and family incomes before FDR correction.

Compared with an unexposed control group with matched CBCL scores and other demographics, the PAE group showed significantly higher intracranial volumes ($t = 3.60$, $p < 0.001$) when controlling for age and sex.

Table S2. Group differences on the CBCL scores and intracranial volumes across visits.

Groups	Measures	95% CI	P value	Cohen's d
PAE - Tobacco	Intracranial volumes	[31527.36, 109582.52]	<0.001	0.7
PAE - CBCL-matched control	Intracranial volumes	[28006.71, 97442.20]	<0.001	0.3
PAE - Tobacco	Anxious/Depressed	[-1.16, 1.66]	0.728	0.10
	Withdrawn/depressed	[-0.98, 1.91]	0.526	0.10
	Somatic complains	[-1.91, 1.24]	0.673	0.10
	Social problems	[-1.47, 0.84]	0.596	0.20
	Thought problems	[-1.25, 1.72]	0.756	0.00
	Attention problems	[-0.24, 2.39]	0.110	0.10
	Rule break	[-1.48, 0.76]	0.531	0.30
	Aggressive	[-1.22, 1.13]	0.938	0.10
	Internalizing	[-2.17, 3.22]	0.704	0.00
	Externalizing	[-2.06, 2.98]	0.720	0.20
	Total problems	[-2.82, 2.98]	0.956	0.10
	DSM5_Depression	[-0.97, 2.08]	0.474	0.00
	DSM5_Anxiety	[-1.40, 1.61]	0.889	0.00
	DSM5_Somatic	[-2.46, 0.92]	0.371	0.10
	DSM5_ADHD	[-0.95, 1.55]	0.638	0.00
	DSM5_Oppositional-defiant	[-1.01, 1.56]	0.678	0.10
	DSM5_Conduct	[-1.88, 0.68]	0.356	0.30
	Sluggish cognitive tempo	[-0.42, 2.30]	0.175	0.10
	Obsessive compulsive	[-0.82, 1.93]	0.430	0.10
	Stress	[-1.33, 1.47]	0.922	0.00
